# The Italian Network for Tumor Biotherapy (NIBIT): Getting together to push the field forward

**DOI:** 10.1186/1479-5876-6-8

**Published:** 2008-02-12

**Authors:** Michele Maio, Hugues JM Nicolay, Paolo Ascierto, Filippo Belardelli, Roberto Camerini, Mario P Colombo, Paola Queirolo, Ruggero Ridolfi, Vincenzo Russo, Lucia Anzalone, Ester Fonsatti, Giorgio Parmiani

**Affiliations:** 1 Division of Medical Oncology and Immunotherapy, Department of Oncology, University Hospital of Siena, Istituto Toscano Tumori, Siena, Italy; 2 Cancer Bioimmunotherapy Unit, Department of Medical Oncology, Centro di Riferimento Oncologico, Istituto di Ricovero e Cura a Carattere Scientifico, Aviano, Italy; 3 Unit of Clinical Immunology, Istituto Nazionale Tumori "Fondazione Pascale" Naples, Italy; 4 Department of Cell Biology and Neurosciences, Istituto Superiore di Sanità, Rome, Italy; 5 Clinical Research Unit III, Sigma Tau SpA, Rome, Italy; 6 Immunotherapy and Gene Therapy Unit, Department of Experimental Oncology, Fondazione Istituto Ricovero e Cura a Carattere Scientifico Istituto Nazionale dei Tumori, Milan, Italy; 7 Department of Medical Oncology A, National Institute for Cancer Research, Genova, Italy; 8 Immunotherapy and Somatic Cell Therapy Unit, Istituto Scientifico Romagnolo per lo studio e la cura dei tumori, Forlì, Italy; 9 Cancer Gene Therapy Unit, Department of Oncology, Scientific Institute S. Raffaele, Milan, Italy; 10 Department of Oncology, San Raffaele Scientific Institute, Milan, Italy

## Abstract

As for a consolidated tradition, the 5^th ^annual meeting of the Italian Network for Cancer Biotherapy took place in the Certosa of Pontignano, a Tuscan monastery, on September 20–22, 2007. The congress gathered more than 40 Italian leading groups representing academia, biotechnology and pharmaceutical industry. Aim of the meeting was to share new advances in cancer bio-immunotherapy and to promote their swift translation from pre-clinical research to clinical applications. Several topics were covered including: a) molecular and cellular mechanisms of tumor escape; b) therapeutic antibodies and recombinant constructs; c) clinical trials up-date and new programs; d) National Cooperative Networks and their potential interactions; e) old and new times in cancer immunology, an "amarcord". Here, we report the main issues discussed during the meeting.

## Introduction

**Figure 1:**
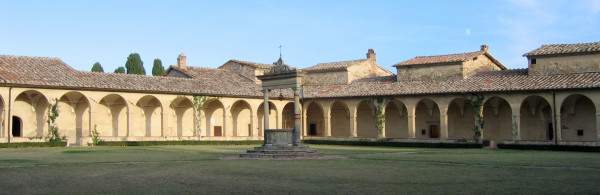
The Main Cloister of the Certosa of Pontignano (Siena, Italy).

The meeting took place in a monastery built by the Carthusian Order on August 1343, located on the border between the town-states of Florence and Siena. The Certosa of Pontignano preserved through the ages its original atmosphere as an oasis of peace (Fig. 1). Nowadays, the monastery is an University guesthouse where the annual symposium of the NIBIT (acronym for the Network Italiano per la Bioterapia dei Tumori – Italian Network for Tumor Biotherapy) is organized. Tuscan landscape and culinary specialties provided a peace of mind and body that helped to reach the event goals.

The increasing knowledge of the molecular mechanisms involved in neoplastic transformation, of the biology of cancer cells, and of the immunological mechanisms regulating tumor-host interactions allows to identify and to apply novel and eventually more effective bio-immunotherapeutic strategies in cancer patients [1].

However, for their optimal clinical development, and for their swift translation from the laboratory to the clinical setting, these new therapeutic approaches require a tight cultural and operative interaction among different professionals involved in the clinical, regulatory and industrial fields [2,3]. Thus, a shared effort is mandatory in order to evaluate with due appropriateness, rapidity and scientific rigor the biological and clinical efficacy of novel treatments that become available. Nevertheless, while cancer bio-immunotherapy is consolidating its role as an additional and powerful option in the comprehensive treatment of cancer patients, it raises new and specific procedural, ethical, and legal challenges to its broader clinical application. Therefore, the efficient clinical development and application of new modalities of cancer bio-immunotherapy is often difficult for large research entities and in most cases impossible for small research units [4].

On these premises, and to cope with a need strongly felt by the Italian academic, industrial and regulatory community, the NIBIT was created in 2004.

### Main objectives of the NIBIT are

a) To promote and foster a stronger scientific and operative interaction among professionals belonging to Academia, Biotech/Pharmaceutical Industry, Regulatory Bodies;

b) To develop innovative, multi-center clinical studies of cancer bio-immunotherapy at national level;

c) To set-up initiatives to inform patients about potentials and limitations of cancer bio-immunotherapy and on ongoing clinical trials [5].

### Scientific welcome to the attendees

In his scientific welcome address, **Lucio Luzzatto **(University of Florence), Scientific Director of the Istituto Toscano Tumori, made an excursus on scientific achievements in the fields of human immunogenetics and cancer immunology that are providing the molecular basis and the scientific rationale to implement novel and eventually more effective immunotherapeutic strategies in cancer patients.

**Molecular and cellular mechanisms of tumor escape **(Chairs: Paola Zanovello and Paola Nisticò)

The presentation of **Francesca Fallarino **(University of Perugia) focused on *"Tumor escape from effector T cells" *and the immunoregulatory enzyme indoleamine 2,3 dioxygenase (IDO). IDO has been associated with a wide variety of human cancers because of its ability to potentiate suppressing regulatory T-cells (T-regs) functions through the catabolism of the essential aminoacid tryptophan. Additionally, it has been recently demonstrated that IDO acts not only as an effector mechanisms of T-regs but also represents a way to generate new T-regs. These findings underscore the importance of the clinical development of IDO inhibitors for suppressing cancer progression [6].

**Susanna Mandruzzato **(University of Padua) presented her experience in *"Myeloid-derived suppressor cells: from mouse to humans"*. Myeloid-derived suppressor cells (MDSC) represent a population of myelo-monocytic cells lacking the markers of mature myeloid cells and expressing both Gr-1 and CD11b in mice. MDSCs bear a high potential to suppress immune responses both *in vitro *and *in vivo*. Mandruzzato showed that even if human MDSC equivalents are not entirely known, granulocyte subpopulations might be involved [7].

Epigenetic modifications generate heritable changes in the expression of single genes or patterns of genes not based on structural changes of the DNA sequence. **Luca Sigalotti **(Centro di Riferimento Oncologico, Aviano) in his presentation *"Epigenetic intervention in tumor escape mechanisms" *demonstrated that hypomethylating agents can modulate the expression of Cancer Testis Antigens and their presentation on the cell surface of neoplastic cells, as well as HLA class I antigens, leading to a more efficient recognition of melanoma cells by cytotoxic T cells (CTL) [8].

**Licia Rivoltini **(Istituto Nazionale Tumori, Milan) discussed on *"Myeloid suppressor cells in melanoma patients"*, reporting that microvesicles or exosomes released by tumor cells and purified from patients' plasma induce CD14+ HLA-DRlow cells with TGF-β-mediated suppressive activity. Therefore, blocking their release by neoplastic cells could improve immune recognition of cancer cells [9].

Tumor-released factors targeting nuclear receptors of dendritic cells (DC) impair DC migration. Along this line, **Eduardo J. Villablanca **(San Raffaele Scientific Institute, Milan) discussed on *"Impairment of DC migration by tumor-released factors targeting DC nuclear receptors" *pointing out that the expression of chemokine receptor CCR7, that guides mature DC from peripheral tissues to lymphoid compartments, is inhibited on DC by soluble factors released from 50% of the melanoma cells investigated *in vitro*.

### Keynote lecture

In his keynote lecture: *"Immune cells within tumor microenvironment: fewer pros than cons"*, **Mario Paolo Colombo **(Istituto Nazionale Tumori, Milan) focused on important implications of the complex of mutual and dynamic relationships occurring between tumor and inflammatory or stromal cells. Evidences exist that tumor progression is supported rather than inhibited by inflammation, and that tumor development is related to the phenotype, function and distribution of inflammatory cells within the tumor mass. Along this line, he provided insights on the potential impact of therapeutic strategies aimed at modifying the population of tumor infiltrating cells [10].

**Antibodies and recombinant constructs **(Chairs: Enrico Proietti and Michele Guida)

The presentation by **Raffaella Giavazzi **(Mario Negri Institute, Milan) on *"Angiogenetic targets and combination therapies" *provided evidence that combination regimens, including two or more biological-vascular targeted inhibitors and conventional therapy, are more promising compared to those based on the use of a single anti-angiogenetic agent. Giavazzi also remarked that the pharmacological delivery schedule and the time/sequence should be carefully evaluated for the optimal administration of these combination treatments [11].

**Angelo Corti **(San Raffaele Scientific Institute, Milan) discussed the *"Targeting tumor endothelium by NGR-cytokines construct(s)"*. He described a therapeutic approach based on the coupling of cytokines with peptides that contain NGR, RGD or isoDGR domains, and that recognize receptors expressed by angiogenetic vessels [12]. Bell-shaped dose response curves have been observed with NGR-TNF, NGR-TNF/EMAP-II and IFN-γ-NGR. Furthermore, Corti presented ongoing and programmed clinical trials of Phase I and Phase II with NGR-hTNF, utilized alone or in combination with chemotherapy.

**Anna Bagnato **(Regina Elena Cancer Institute, Rome) presented data on *"Molecular targeting of endothelin B receptor (ET*_*B*_*R) in melanoma" *demonstrating that ET_B_R expression is associated with melanoma progression, and that blocking of ET_B_R by antagonists results in tumor growth inhibition and in a reduced expression of endothelial markers. Thus, new strategies using specific ET_B_R antagonists could provide an improved approach to the treatment of melanoma. Along this line, clinical trials with a dual (ET_A _and ET_B_) endothelin receptor antagonist (Bosentan) that acts by competitively and specifically binding to ET_A _and ET_B _receptor sites in the endothelium and vascular smooth muscle, are ongoing in metastatic melanoma patients [13].

In her presentation *"Targeting Her2 in breast cancer" ***Elda Tagliabue **(Istituto Nazionale Tumori, Milan) showed that Antibody-Dependent Cellular Cytotoxicity (ADCC) constitutes a mechanism involved in the therapeutic activity of Trastuzumab; however, quantity and lytic efficiency of CD16+CD56+ lymphocytes are major factors for the ADCC heterogeneity observed in treated patients [14]. Additionally, the efficacy of Trastuzumab also depends on its cytostatic effect. An Italian multi-center retrospective and prospective study, called DEMETRA, on 444 women with metastatic HER2-positive breast carcinoma treated with Trastuzumab has been opened to assess clinical responses, metastatic sites with better susceptibility to treatment, the best chemotherapeutic combination(s) with Trastuzumab and its toxic effects.

**National Cooperative Networks: potential interactions?**(Chairs: Ruggero Ridolfi and Vincenzo Russo)

Cancer represents more than 200 diseases, and for its best cure it clearly requires a multi-disciplinary knowledge and approach. This session was based on possible and required interplays between Italian Networks for cancer treatment. Four Italian groups were invited to explain the aim of their associations and to foresee potential pre-clinical and clinical interactions with the NIBIT: **Piero Picci **(Bologna) for the *"Italian Sarcoma Group" *[15], **Paolo Ascierto **(Naples) for the *"Italian Melanoma Intergroup" *[16], **Paolo Pedrazzoli **(Milan) for the *"Italian Bone Marrow Transplant Group" *[17] and **Nicoletta Zilembo **(Milan) for the *"Italian Trials in Medical Oncology" *[18]. The main goals of these interactions are to make uniform the clinical diagnosis and therapeutic protocols, improve patient wellness, foster collaboration between national and international Institutions, provide meeting opportunities to exchange and teach advances in cancer treatments.

**Old and new times in cancer immunology: an "amarcord" **(Chairs: Mario Paolo Colombo, Paolo Dellabona and Pierluigi Lollini)

Among the aims of the meeting, one was to provide an overview in specific areas of cancer research in the last two decades. It was deemed important to deliver lectures for the next generation of cancer researchers and clinicians, to foster the understanding of ongoing clinical trials and research projects destinations. Six senior lecturers realized this "tour de force" to explain the evolution of cancer care and their personal contribution into it.

**Giorgio Parmiani **(San Raffaele Scientific Institute, Milan) delivered the talk *"From tumor immunology to immunotherapy: the ups and downs of the last two decades"*. In particular, the presentation was focused on immune-surveillance, tumor specific transplantation antigens, IL-2 cytokine therapy, human tumor antigens and vaccination with peptides/proteins [19].

**Pier Giorgio Natali **(Regina Elena Cancer Institute, Rome) with the presentation *"Tumor immunophenotyping: a two-way ticket between laboratory and the clinic" *argued on the necessity to continue the expansion of the banks of human tissues to further understand cancer biology, and to finally achieve the molecular classification by gene expression profiling of all human cancer types [20].

The presentation by **Sergio Romagnani **(University of Florence) *"Effectors and regulatory T cells in man" *showed how CD4+ cells are important in cancer treatment, through their functional role as effector, helper and regulatory cells. He also demonstrated that human Th17 cells, a subset of interleukin 17-producing T helper cells, that could play a key role in autoimmune disease, are different from mouse Th17 cells [21].

**Alberto Amadori **(University of Padua) with *"B cell activation: deeds, misdeeds, and serendipity" *explained how interactions between B and T cells are crucial for the immune system. Then, he talked on the role of hypoxia in human lymphomas, providing evidence that it inhibits macrophages and monocytes migration and up-regulates CXCR4 surface expression in B-cell lymphomas [22].

The presentation by **Lorenzo Moretta **(University of Genoa) *"The NK in cancer: A new tool for cancer therapy?" *provided insights on a new approach for the treatment of acute leukaemia by using NK cells-based therapy. He demonstrated that the size of the alloreactive NK cell subset parallels the degree of NK cytotoxicity against leukaemic cells [23].

**Guido Forni **(University of Turin) with *"Mind the mouse: drawbacks and illuminations by experimental models" *pointed out the still controversial issue of whether genetically engineered mice could be an appropriate and reliable model for cancer immunity [24].

### Keynote lecture

**Paolo Bruzzi **(National Institute for Cancer Research, Genoa) opened the session on clinical studies delivering the keynote lecture: "*Methodological challenges in trials of novel anticancer therapies*". A clinical study in cancer patients should be designed remembering Sackett's words: "Most controversies on the interpretation of the results of clinical trials derive from disagreements or ambiguities on the aims of the trial". In fact, the identification of its aims is the most important phase of a clinical trial design. Clinicians, biologists and statistician should work together to specifically define the primary endpoints of a clinical study, in order to not misinterpret its results only because they do not hit the bull's eye [25].

**Clinical studies: Up-date and New Programs **(Chairs: Giorgio Parmiani and Michele Maio)

The presentation by **Enrico Proietti **(Istituto Superiore Sanità, Rome) on "*Chemo-immunotherapy: an advanced approach in melanoma" *provided evidence that chemo-immunotherapy induces a plethora of immunomodulating changes in PBMCs. Microarray and Real Time PCR data, on PBMCs of tumor-resected patients treated with Dacarbazine (DTIC), demonstrate that treatment modulates the levels of expression of homeostatic cytokines like IL-2 and IL-15 [26].

**Roberto Camerini **(Sigma Tau, Rome) with *"Thymosin alpha-1 in metastatic melanoma" *described a large first-line, randomized, dose-finding, phase II Study on Thymosin alpha 1 (Tα1) plus DTIC, with or without Interferon alpha (IFN-α), vs. DTIC plus IFN-α in stage IV melanoma patients. This study showed that the best candidate regimen for a future phase III trial is DTIC + Thymosin 3.2 mg since, opposite to the DTIC + IFN-α control arm, it reached the number of tumor responses required to reject the null hypothesis (at least 9 responses) [27].

Based on the mouse model data demonstrating that Salmonella infected tumor cells are killed by anti-salmonella specific T cells, **Francesco Ferrucci **(European Institute of Oncology, Milan) presented a *"Pilot phase II study of a combined immunotherapy protocol based on oral vaccination and direct intratumoral injection of Salmonella Typhi in metastatic cutaneous melanoma patients"*. The study is ongoing and nine patients have been enrolled so far [28].

**Barbara Capaccetti **(Pfizer Oncology, Rome) presented on "*CTLA-4 in metastatic melanoma*", a Pfizer designed phase I study, then moved to phase II, in which advanced and high risk melanoma patients were treated with the anti-CTLA-4 monoclonal antibody (Tremelimumab) at varying dose levels. Primary objective of this study was to assess safety and tolerability of the drug. Results show that not only Tremelimumab is manageable for safety profile and that the schedule of administration is very suitable, but, most important, median survival was 15,9 months for all dose group combined [29].

**Cosimo Paga **(Bristol-Myers Squibb, Rome) also reported on "*CTLA-4 in metastatic melanoma*". Bristol-Myers Squibb (BMS) sponsored several clinical trials with an anti-CTLA-4 monoclonal antibody (Ipilimumab) administered as monotherapy or in combination with DTIC in melanoma patients. It has been observed that the administration of Ipilimumab, alone or in combination with DTIC, increases the overall survival of patients vs. DTIC alone. For safety profile, adverse events are characteristic of Ipilimumab treatment and probably result from its mechanism of action; however, they are usually reversible, linked to drug exposure, and appear to correlate with clinical response [30].

**Luana Calabrò **(University Hospital of Siena) presented a new study on the use of: "*CTLA-4 in malignant mesothelioma*". Malignant mesothelioma (MM) is a neoplasm endowed by a very poor prognosis, whose incidence has increased steadily and progressively over the last 30 years. As there is no standard treatment, MM can be considered a "model disease" to explore new therapeutic regimens. Pre-clinical and clinical studies have shown that MM patients can mount a tumor-specific immune response; however, this immune activation does not result in tumor rejection, likely because the mesothelioma microenvironment comprises cytokines and T-reg cells that effectively suppress immune responses. Thus, a second line phase II clinical trial with Tremelimumab has been designed to treat patients with unresectable MM. Primary aim of the study will be to assess the rate of objective clinical responses (complete and partial responses); while secondary end points will be to define toxicity profile, to assess the progression free survival, and to evaluate qualitative and quantitative changes in cellular and humoral responses [31].

**Francesco Leone **(University of Turin) described the upcoming trial: "*CTLA-4 in pancreatic cancer*". Blocking of CTLA-4 with anti-CTLA-4 antibodies may represent a way to break the peripheral immunologic tolerance in pancreatic cancer patients and to restore an adequate immune response against the tumor. The trial will be conducted as a prelude to a planned phase II trial of anti-CTLA-4 antibody in combination with gemcitabine that has shown immune-enhancing effects. Primary endpoint of the trial is to define safety and maximum tolerated dose of the drug.

In his presentation "*Adjuvant vaccination in prostate cancer*" **Andrea Marrari **(Istituto Nazionale Tumori, Milan) summarized an ongoing trial in which multiple peptides derived from the prostatic associated-antigen (PSA) are administrated in association with Montanide ISA51 to patients with relapsing disease after surgery or radiotherapy. The 16 enrolled patients have completed the first cycle of vaccination, whereas only 9 completed the entire schedule. Three patients developed an immunological response to the vaccine, while 5 of them showed a stabilization or reduction of PSA levels [32].

**Federica Danzi **(Glaxo Smith-Kline, Italy) reported on: "*MAGE-3 vaccination in lung cancer*". MAGE-3 is an immunogenic Cancer Testis Antigen whose expression is associated with a poor prognosis in lung cancer. A recombinant MAGE-3 protein was administered in phase I/II clinical trials in non-small cell lung (NSCL) cancer patients. Based on the promising clinical results obtained, a multi-center double blind randomized phase III study called **M**AGE-A3 as **A**djuvant non-small cell lun**G **cancer**R I**mmuno**T**herapy (MAGRIT) was designed. 2270 patients with MAGE-3 positive NSCL cancer of stage IB, II or IIIA will be enrolled after surgery in four arms receiving: i) platinum-based chemotherapy and placebo; ii) platinum-based chemotherapy and MAGE-3 protein; iii) placebo; iiii) MAGE-3 protein. Primary endpoint of the study is the assessment of the disease free-survival.

**Luigi Aurisicchio **(Merck, Rome) described a novel mouse model for the: "*Enhancement of cancer vaccines efficacy*". A vaccination study in Balb/neuT mice demonstrated that the combination of CEA/HER2 DNA-based vaccine with an immuno-modulator (i.e., IMO-1, a TRL9 agonist) enhanced the immune response in mice, retarding and/or blocking tumor growth. These evidences once again strengthened the importance of immunomodulatory agents in cancer vaccinations. The results obtained from this pre-clinical study could help to redesign disappointing past clinical studies based on DNA vaccination.

**Paola Queirolo **(National Institute for Cancer Research, Genoa) presented the study: *"High dose interferon-α2b (HDI) or PEG-Intron in combination with a multipeptide-based vaccine in metastatic (M1a, M1b AJCC) melanoma patients. A phase II randomized NIBIT study"*. This is the first clinical trial designed within the NIBIT. The study comprises three arms: the first in which metastatic melanoma patients will receive HDI plus a multipeptide vaccine (i.e., gp100, NY-ESO-1 and survivin derived peptides emulsified in Montanide); the second in which patients will be treated with pegylated interferon-α2b (PEG-Intron) plus the multipeptide vaccine; the third in which patients will receive PEG-Intron alone. The primary aims of the study are to evaluate the feasibility of the procedure and to investigate tumor-specific humoral and cellular immune responses [33].

**Lorenzo Pilla **(Istituto Nazionale Tumori, Milan) presented preliminary findings on the ongoing trial "*Multipeptide-based vaccination in melanoma*". Currently, no validated therapeutic regimens are available for stage II/III melanoma patients who underwent radical surgery and are at high risk of disease relapse. On this basis, a phase II randomized trial is being carried out to determine the immunologic and clinical effect of a vaccine based on immunogenic peptides from MART-1:26–35 (27 L), gp100: 209–217 (210 M), NY-ESO-1: 157–165 V and survivin-1: 96–104, administered in combination with Montanide ISA 51 and IL-2 as adjuvant. Preliminary results show that vaccination increased the levels of CTL specific for all the antigenic peptides utilized in the study. An analysis of the T-reg population is ongoing to see its variation in the blood and vaccine-draining lymph nodes.

**Gaetano Finocchiaro **(Fondazione IRCCS Istituto Neurologico Besta, Milan) delivered the talk on "*Vaccination with cancer stem cells (CSC) in glioblastoma (GB)*". CSC were derived from GB and grown *in vitro *with specific growth factors (i.e., EGF and bFGF) to form neurospheres. *In vivo *experiments demonstrated that these neurospheres have the potential to express glial and/or neuronal markers and form highly infiltrating GB into the brain of immune-deficient mice. Furthermore, the use of DC loaded with lysates from GB cells or neurospheres as therapeutic vaccines can arrest tumor growth and retard its relapse in mice grafted with murine GB cells. A phase I clinical trial, using autologous DC loaded with lysate from patient's tumor (DCVax^®^-Brain manufacturing), was administered after surgery and radiotherapy, concurrent with chemotherapy to 18 patients with GB multiform. As the vaccine did not present toxicity and significantly extended progression free survival and increased overall survival, the clinical trial moved to phase II [34].

**Marco Bregni **(San Raffaele Scientific Institute, Milan) reported on: "*Intraperitoneal therapy with dendritic cells in ovarian cancer*". The induction of tumor cell death with chemotherapeutic agents can induce a tumor-specific immune response if appropriate adjuvant signals are delivered with an appropriate timing. Ovarian cancer represents a suitable model for intraperitoneal therapy with DCs, as in 80% of cases the disease is confined to the peritoneum. Along this line, a proposal for a pilot study in advanced ovarian cancer patients with recurrent disease was presented. DCs and natural killer/lymphokine-activated killer (NK/LAK) cells, derived from the enrolled patients and activated *in vitro *for 6 days, will be administered 48 hours after standard chemotherapy. Primary objective of this study will be the assessment of the feasibility and safety of the procedure. Secondary endpoints will be the definition of specific anti-tumor immune responses, clinical response rate and time to progression free-survival of patients.

**Franco Venanzi **(University of Camerino) presented on "*Do tumor endothelial marker 8 (TEM8) gene-expression levels impact clinical outcome in dendritic cells-based cancer vaccination?*". TEM8, a membrane anthracis receptor, is selectively over-expressed on tumor cells, tumor endothelium and in DCs maturated in the presence of tumor cells. A retrospective study in melanoma patients vaccinated with DCs loaded with autologous tumor lysate showed that increased levels of TEM8 expression in DCs vaccine preparations correlate with disease progression [35]. These preliminary findings suggest that high levels of TEM8 expression might represent an exclusion criterion for DC-based cancer vaccination.

**Daniela Montagna **(University of Pavia) talked about: "*Evaluation of feasibility and safety of the infusion of autologous tumor-reactive T cells in sarcoma patients*". The phase I study presented was conducted in sarcoma patients refractory to chemotherapy or relapsing during chemotherapy. The protocol was based on *ex vivo *induction of allogeneic or autologous CTL lines activated with tumor cells and then re-infused to the patient. The study showed that the treatment is well tolerated and that it is also capable to induce stabilization of disease and to prolong overall survival. Optimization of this immunotherapeutic strategy is currently under investigation [36].

## Conclusion

During the 5^th ^meeting of the NIBIT, scientists representing academia, biotechnology and pharmaceutical industry put together their ideas, results and their good will to make real translational cancer biotherapy. Here, we insist on the necessity to continue and strengthen this collaboration both nationally and internationally, [37] and we invite basic scientist, clinical researchers and industries to contact us and join the network to overstep obstacles in cancer care improvement [38].

## Competing interests

Roberto Camerini, Director of the Clinical Research Unit III of Sigma Tau SpA (Rome, Italy), as an employee of Sigma Tau SpA has been involved in designing and conducting the clinical study *"Thymosin alpha-1 in metastatic melanoma" *which was presented during the meeting and is reported in the present article. Other authors declare that they have no competing interests.

## Authors' contributions

All authors read and approved the final manuscript.
